# Synergistic Inhibition of Angiogenesis by Artesunate and Captopril *In Vitro* and *In Vivo*

**DOI:** 10.1155/2013/454783

**Published:** 2013-10-08

**Authors:** Benjamin Krusche, Joachim Arend, Thomas Efferth

**Affiliations:** Department of Pharmaceutical Biology, Institute of Pharmacy and Biochemistry, Johannes Gutenberg University, Staudinger Weg 5, 55128 Mainz, Germany

## Abstract

Inhibition of angiogenesis represents one major strategy of cancer chemotherapy. In the present investigation, we investigated the synergism of artesunate and captopril to inhibit angiogenesis. Artesunate is an antimalarial derivative of artemisinin from the Chinese medicinal plant, *Artemisia annua* L., which also reveals profound anticancer activity *in vitro* and *in vivo*. Captopril is an angiotensin I-converting (ACE) inhibitor, which is well established in Western academic medicine. Both compounds inhibited migration of human umbilical vein endothelial cells (HUVECs) *in vitro*. The combination of both drugs resulted in synergistically inhibited migration. Whereas artesunate inhibited HUVEC growth in the XTT assay, captopril did not, indicating independent modes of action. We established a chorioallantoic membrane (CAM) assay of quail embryos (*Coturnix coturnix* L.) and a computer-based evaluation routine for quantitative studies on vascularization processes *in vivo*. Artesunate and captopril inhibited blood vessel formation and growth. For the first time, we demonstrated that both drugs revealed synergistic effects when combined. These results may also have clinical impact, since cardiovascular diseases and cancer frequently occur together in older cancer patients. Therefore, comorbid patients may take advantage, if they take captopril to treat cardiovascular symptoms and artesunate to treat cancer.

## 1. Introduction

Although cancer treatment is multimodal with chemotherapy, radiotherapy, and surgery as classical therapy options and treatment with antibodies as new strategy, the cure from their disease is still not a reality for many cancer patients. The development of resistance and the severe side effects by chemo- and radiotherapy on the one hand and the inability to remove disseminated single tumor cells and micrometastases on the other hand lead to unsatisfactory treatment results and high mortality. Since decades it has been suggested to supplement established treatment modes by approaches from complementary and alternative medicine. While conventional academic medicine (Western medicine) is reductionistic, complementary medicine and alternative medicine propose holistic approaches to cure patients [1]. 

Although integrative oncology succeeded a lot in combining the best of both “worlds” during the past two decades, there is still much debate about the scientific evidence for the efficacy and safety of complementary and alternative medicine [2, 3]. Integrative oncology comprises several aspects from complementary and alternative medicine, for example,

- psychological approaches to improve mental conditions of cancer patients after diagnosis and during therapy such as mind-body therapy and music therapy [4, 5];
- treatment of pain by acupuncture, acupressure, massage, herbal teas, and so forth [6];
- dietary measures either to prevent cancer and influence the course of the disease after chemotherapy, radiotherapy, and surgery [7–9];
- tumor reductive therapy by medicinal herbs or isolated natural compounds that inhibit cancer growth either alone or by synergistic interactions with established anticancer drugs [10–14].

In the present investigation, we focused on the last of these four aspects and analyzed the synergistic interaction of a compound derived from Chinese medicine (artesunate) and a compound established in Western academic medicine (captopril). *Artemisia annua* L. (Sweet Wormwood, *qinghao*) is an herb used in traditional Chinese medicine to treat fever and chills [15, 16]. In the 1970s, the active principle of the plant, artemisinin, has been identified as an antimalarial sesquiterpene [17]. In the 1990s, we and others discovered the profound anticancer activity of artemisinin-type compounds [18–20]. As artemisinin is poorly water soluble, its derivative artesunate is better suited for pharmacological studies. Artesunate and artemisinin are not only cytotoxic towards cancer cell lines *in vitro*, but also exert antitumor activity against human xenograft tumors in nude mice [21–23]. The activity of artesunate against uveal melanoma and cervical carcinoma in patients has been reported [24, 25]. As most natural products, artesunate is a multifactorial compound that attacks cancer cells by various mechanisms, including inhibition of angiogenesis, induction of DNA damage, cell cycle arrest, apoptosis, and others [21, 26–32].

Captopril has been established in clinical practice since many years as angiotensin-I-converting enzyme (ACE) inhibitor to treat cardiovascular diseases such as congestive heart failure and arterial hypertension [33]. Although, captopril is a chemical relative of a poisonous compound from the pit viper *Bothrops jararaca* WIED [34], captopril and other ACE inhibitors are usually not regarded as natural product derivatives and belong to the established pharmacopoeia of Western academic medicine. Interestingly, captopril acts not only on the cardiovascular system, but also inhibits tumor growth by inhibition of tumor neoangiogenesis [35, 36]. 

The generation of new blood vessels occurs physiologically during embryogenesis, but also under pathophysiological conditions, for example, cancer, rheumatoid arthritis, and psoriasis [37]. During angiogenesis, blood vessels are encased by single cell layers of endothelial cells, which generate a barrier between blood flow and surrounding tissues. Alongside gradients of angiogenic growth factors such as the vascular endothelial growth factor (VEGF), endothelial cells proliferate and migrate into the free space, thereby forming new capillaries. Angiogenesis is a crucial step in cancer growth, since blood supplies the tumor with nutrients and oxygen on the one hand and dramatically increases the probability for metastasis on the other hand [38]. Therefore, therapeutic antibodies and synthetic small molecules have been developed in the past years to specifically inhibit tumor angiogenesis [39]. Despite increases in progression free survival, no major benefit to overall survival was described for the currently approved antiangiogenic drugs due to acquired resistance [38]. Therefore, there is an urgent need for novel drugs and treatment strategies.

The aim of the present investigation was to explore the combination treatment of artesunate and captopril concerning possible synergistic interaction on angiogenesis. For this reason, we developed a modified chorioallantoic membrane (CAM) assay to study vascularization of quail eggs *in vivo* with and without drug treatment. For *in vitro* analyses, the cytotoxicity of artesunate and captopril was determined by XTT assay using human umbilical vein endothelial cells (HUVEC). Inhibition of cellular migration *in vitro* by the two compounds was assessed by a HUVEC migration assay.

## 2. Material and Methods

### 2.1. Drugs and Chemicals

Artesunate was obtained from Saokim Ltd. (Hanoi, Vietnam) and captopril from Fagron (Barsbüttel, Germany). All other chemicals were of *pro analysi* quality and were purchased from Merck (Darmstadt, Germany) and Sigma-Aldrich (Taufkirchen, Germany).

### 2.2. *Ex Ovo* Cultivation of Eggs

Quail eggs (*Coturnix coturnix* L.) were obtained from *Wachtelzucht* Anne Klein (Steinhagen, Germany) and maintained in an incubator of Bruja (Brutmaschinen-Janeschitz GmbH, Hammelburg, Germany).

**Figure 1:**
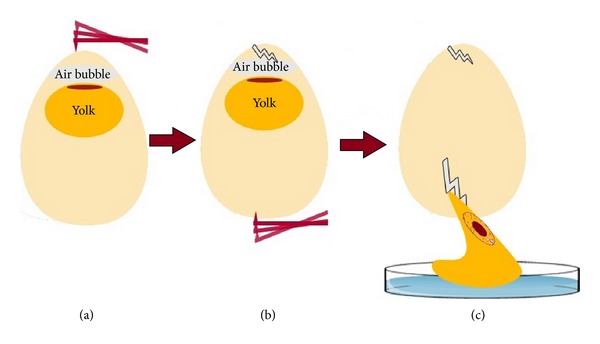
Transfer of fertilized quail eggs to Petri dishes.

Fertilized quail eggs were incubated for 70 h at 38°C and maximum humidity. A special incubator was used which provided a regular turning of the quail eggs. After 70 h of incubation, the eggs were opened. For this purpose, the eggs were placed in a vertical position to guarantee that the embryo float in the upper part of the eggs. Afterwards, a hole was cut into the top of the egg. By rupturing the bottom side of the egg, the complete content of the egg was transferred into a Petri dish. Thereby, it could be guaranteed that first the egg-white flew into the Petri dish followed by the yolk with the embryo on top without exposing the embryo the shock-forces which could damage the vitelline membrane. The procedure is shown in Figure 1. 

### 2.3. Drug Treatment

The explanted embryo was placed in an incubator for 2 h at 38°C to acclimatize to the new ambience. Subsequently, the test substances were placed on the chorioallantoic membrane. A 2% agarose solution was prepared which was placed in a 60°C water bath to prevent preterm solidification of the agarose. The solution was mixed 1 : 10 with the test substance, diluted in DMSO. Ten microliters of the mixed solution was placed on a PVC pistil with a diameter of 3 mm. The agarose pellets got solid after a short time period and were placed on the chorioallantoic membrane after cooling down to room temperature. The Petri dishes with the embryos were placed in the incubator again and incubated at 38°C and maximal humidity for 24 h prior to documentation of the drug effects.

### 2.4. Quantitative Analysis of Vascularization

Imaging of the vascularized eggs was performed using a digital camera with threefold magnification. For illumination, a mercury-arc-lamp was used, which provided a high fraction of blue and UV-light to obtain good contrast values between yolk and vessels. The recorded image section had a size of 3 × 5 mm. Following image acquisition, quantitative analysis was performed using a software routine written in ImageJ-macro language. 

#### 2.4.1. Noise Reduction

To enhance the segmentation results, images were smoothed at the beginning using standard filters. Anisotropic diffusion filtering was used for general smoothing, as it filters strongly in regions without prominent details but does not considerably affect edges. This ensured that even very thin vessels were not removed by the filtering steps, which otherwise can occur, for example, by using a Gaussian-blur filter. As the camera chip produced a lot of white noise, a special filter offered by ImageJ was used to de-speckle the images.

#### 2.4.2. Segmentation

Segmentation of the smoothed images was performed by comparing the intensity values of the pixels in a local neighborhood. The threshold for segmentation was automatically determined using the Bernsen method. This is a local segmentation method which was originally developed to obtain a robust segmentation of vessels on images which were not equally illuminated [40]. The threshold in this method is the mean value of maximum and minimum of the intensities of all pixels in certain neighborhood. Using this threshold the image was binarized.

#### 2.4.3. Analysis of the Total Area of Thin Vessels

There were already some thicker vessels existing in the egg before applying the substances which could strongly affect the results. Therefore, these thicker vessels have to be removed after the segmentation procedure. All vessels with a diameter of more than 15 *μ*m were removed. Therefore, a copy of the image was generated in which all thin objects (<15 *μ*m) were removed. Afterwards, this image was subtracted from the original image obtaining a picture which contained only small capillaries. For quantification, the total amount of black pixels in this image was counted and saved to a file.

#### 2.4.4. Analysis of Branching and Capillary Length

To gain additional information about the vascular system, the original segmented image was used for the analysis of the mean capillary length and the grade of branching. For this reason, the images were skeletonized, so that every vessel was represented as a one pixel thick structure in the image. Now, it was possible to determine the junctions and the vessel lengths using simple filter techniques. Black pixels with less than two neighboring pixels were defined as endpoints of vessels. Pixels having more than two neighboring pixels were junctions and pixels with exact two black neighboring pixels were in the middle of a vessel. These pixels were counted. 

## 3. Results

### 3.1. Establishment of the Quail Egg CAM Assay

**Figure 2:**
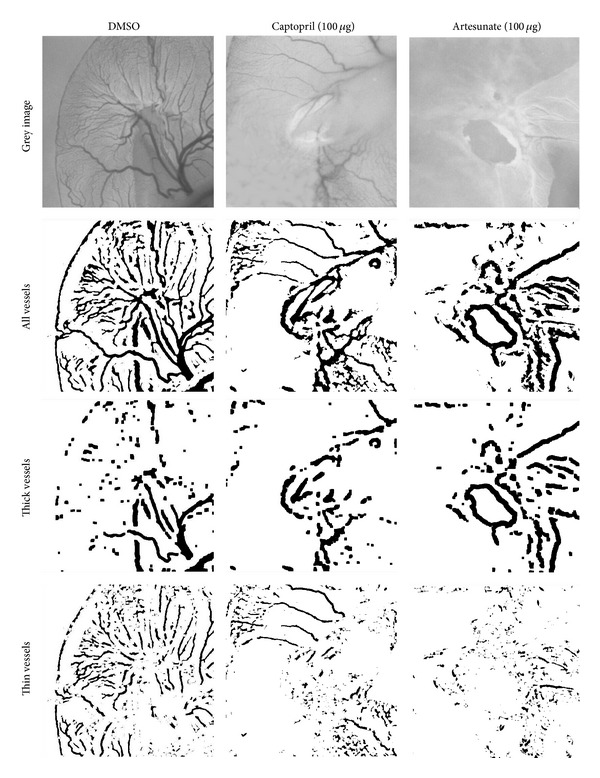
Graphical analysis of antiangiogenic effects. Inhibition of vessel growth in quail eggs maintained for 72 h was observed after incubation with 100 *μ*g captopril or artesunate for 24 h. A decrease in coarse and fine structure of vessels was observed compared to DMSO (negative control).

**Figure 3:**
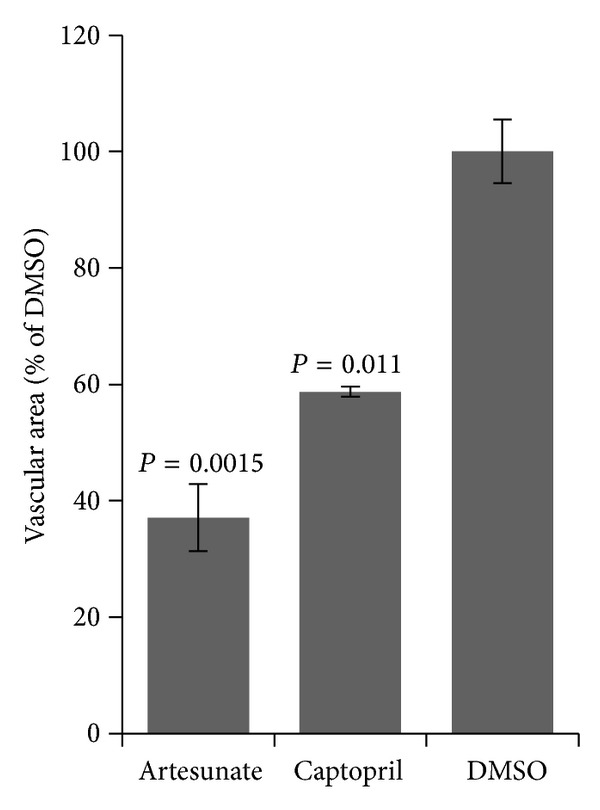
Quantification of vascularization in quail eggs maintained for 72 h was observed after incubation with 100 *μ*g captopril or artesunate for 24 h. Both drugs significantly inhibited vascularization compared to DMSO (negative control) (Student's *t*-test). The areas of fine capillaries were measured by a software routine described in Section 2.

As a starting point, 100 *μ*g artesunate or captopril per 10 *μ*L pellet were applied to chorioallantoic egg membranes. Dimethylsulfoximine (DMSO) was used as negative control. As shown in Figure 2, both drugs caused significant reductions in the vascular surface area. The remaining veins in artesunate-treated eggs were not red in color anymore, indicating that artesunate affects both blood vessel growth and structure. This effect was not observed in captopril-treated eggs. A quantitative analysis of the experiments revealed that both artesunate and captopril significantly inhibited blood vessel formation compared to the negative control, DMSO (Figure 3).

**Figure 4:**
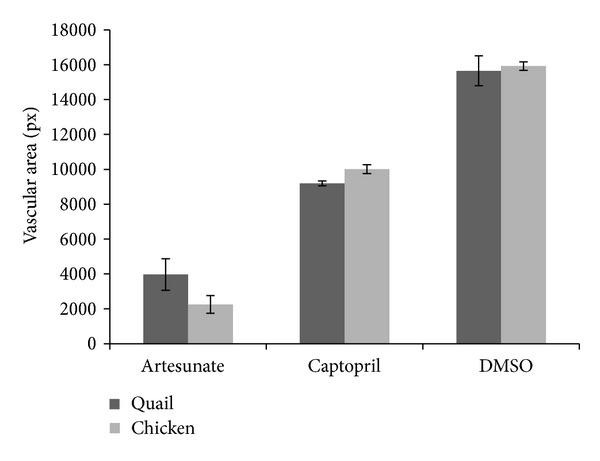
Comparison of inhibition of vascularization in quail and chicken eggs. Eggs were maintained for 72 h and incubated with 100 *μ*g captopril or artesunate for 24 h. The area of fine capillaries was measured by a software routine described in Section 2.

As the CAM assay is more common for chicken than for quail eggs, we compared the results obtained for artesunate or captopril-treated quail eggs with those for chicken eggs. As can be seen in Figure 4, inhibition of vascular areas after treatment with artesunate or captopril was similar for quail and chicken eggs. 

### 3.2. Analysis of Blood Vessel Branching in Quail CAM Assay

**Figure 5:**
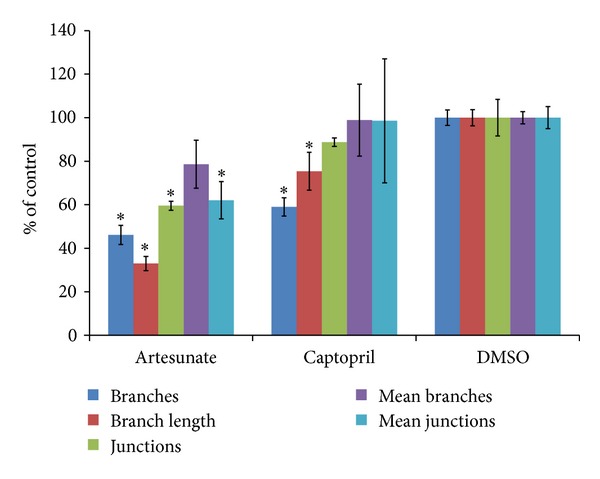
Branching of blood vessels after drug treatment. Data have been normalized as % of untreated control. Statistically significant differences have been calculated by Student's *t*-test. Asterices indicate significance values of *P* < 0.02.

In addition to calculating the vascular areas (Figures 2 and 3), we measured the number and length of the veins as well as the degree of vessel branching (Figure 5). The fraction of branches and the branch lengths in artesunate- or captopril-treated quail eggs significantly differed from the negative control, DMSO (*P* < 0.02, students' *t*-test). The fraction of junctions was significantly lower in artesunate-treated but not captopril-treated eggs compared to DMSO.

### 3.3. Testing of HUVECs in XTT Assay

**Figure 6:**
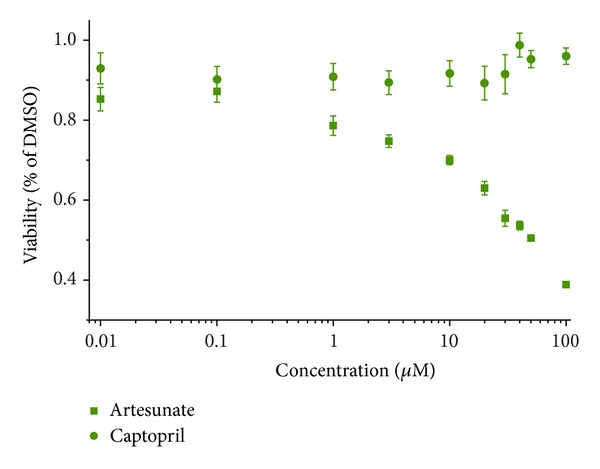
Effect of artesunate and captopril on human umbilical vein endothelial cells (HUVEC). Cells were treated for 72 h with serial dilutions of the drugs and measured by the XTT assay. Artesunate dose-dependently inhibited growth of HUVEC, whereas captopril did not.

 HUVEC cells were treated with artesunate or captopril in a dose range of 0.01 to 100 *μ*M for 72 h and subjected to XTT assay. Although artesunate inhibited the proliferation of HUVEC cells in a dose-dependent manner, captopril did not show any effect over the entire dose range (Figure 6). 

### 3.4. HUVEC Migration Assay

**Figure 7:**
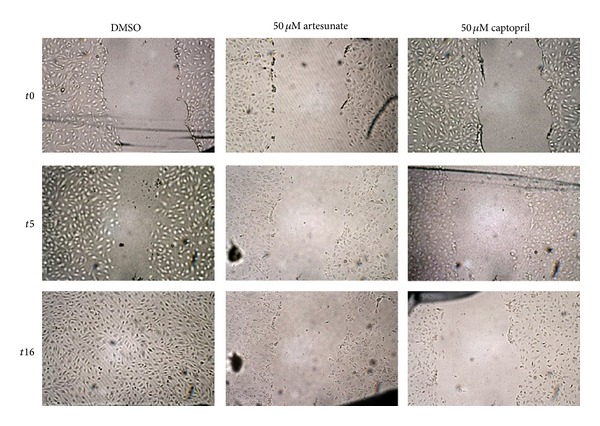
Effect of artesunate and captopril on wound healing of HUVEC monolayers. Artesunate or captopril (each 50 *μ*M) inhibited closing of the wound in a time-dependent manner.

**Figure 8:**
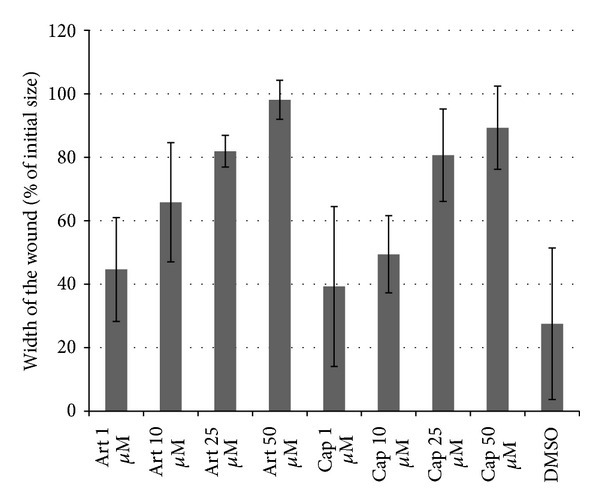
Wound healing in HUVEC monolayers after treatment with artesunate or captopril. The closing of the wound was observed 5 h after scratching. Both drugs caused a dose-dependent inhibition of migration and proliferation of HUVECs.

**Figure 9:**
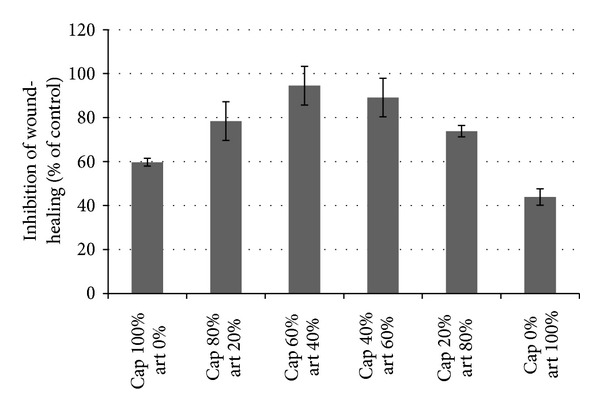
Synergistic effect of artesunate and captopril in HUVEC monolayers *in vitro*. The closing of the wound was observed 5 h after scratching. Both drugs were mixed at different ratios as indicated, 100% mean IC_50_. If the effect is only additional, 50% inhibition is expected. Values above 50% indicate synergistic interaction, which was observed by the combination of both drugs.

As a simple proliferation assay could not show any effect of captopril, a wound-healing assay with HUVEC cells was performed. The wound size decreased in the DMSO-treated negative control in a time-dependent manner, whereas treatment with 50 *μ*M artesunate or 50 *μ*M captopril inhibited the closing effectively even 16 h after the scratch was made (Figure 7). The wound size was measured 5 h after the initial scratch. The IC_50_ values were approximately 10 *μ*M for artesunate and approximately 25 *μ*M for captopril. As a next step, the substances were prepared at their IC_50_ concentrations and mixed in different ratios to observe possible synergistic effects of artesunate and captopril. If the substances would act in an additive manner, 50% inhibition of wound-healing should be expected in all of the different mixing ratios. Synergistic effects would lead to an increased inhibition of wound-healing in the mixed samples. The results are shown in Figure 8. The data are represented as relative inhibition values compared to DMSO-treated cells. The closing of the wound in DMSO-treated cells was considered as no inhibition. The inhibition of wound-healing was increased from 50% in single dose treatments to 100% inhibition in a mixed ratio of 60% captopril and 40% artesunate indicating that artesunate and captopril synergistically interacted in inhibiting wound-healing of HUVEC monolayers *in vitro* (Figure 9).

### 3.5. Synergistic Interaction of Artesunate and Captopril in Quail Egg CAM Assay

**Figure 10:**
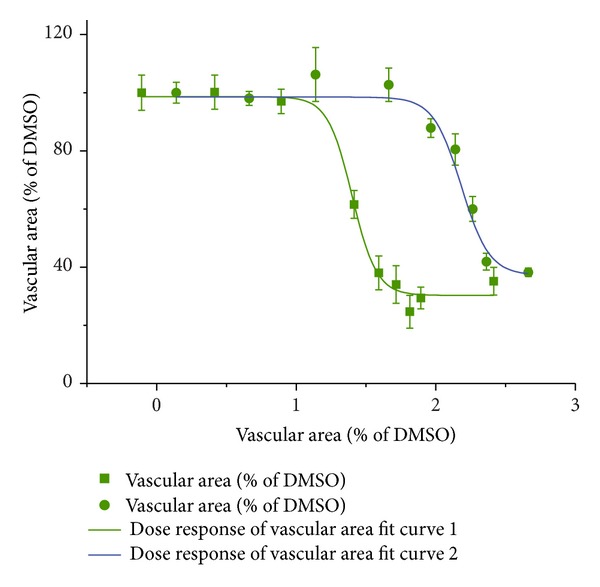
Dose response curves of artesunate and captopril as assayed by the CAM assay. The IC_50_ value for artesunate and captopril were 24.13 nM and 154.66 nM, respectively.

**Figure 11:**
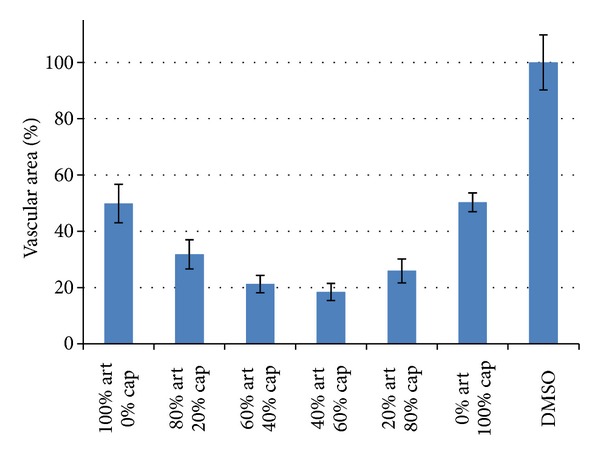
Synergistic effect of artesunate and captopril *in vivo*. Both drugs were mixed at different ratios as indicated. 100% means IC_50_. If the effect is only additional, 50% inhibition is expected. Values below 50% indicate synergistic interaction, which was observed by the combination of both drugs.

To investigate a possible synergism between artesunate and captopril *in vivo*, the IC_50_ values of single doses of both substances have been determined. The obtained vascularization values were fitted as sigmoidal curves and the IC_50_ values were calculated using the Origin Pro 8.0 software (Figure 10). Then, the substances were prepared at their IC_50_ and mixed in different ratios. If the effect of one substance would not be affected by the other drug, the obtained vascular area is expected to be always 50% of the negative control. If the obtained inhibition is lower than 50%, the substances act antagonistic, and if the inhibition is significantly higher than 50%, the substances act synergistically. The results are shown in Figure 11. As expected, single dose treatments with IC_50_ concentrations of either artesunate or captopril led to an inhibition of 50%. Combination treatments with both drugs revealed much stronger inhibition with a maximum at a mixing ratio of 40 : 60 (artesunate : captopril) and an inhibition of 83%. This indicates that artesunate and captopril synergistically inhibited angiogenesis *in vivo*.

## 4. Discussion

### 4.1. Establishment of a Quail Egg CAM Assay

We showed the feasibility of an *ex ovo* approach based on quail eggs to study the effect of antiangiogenic substances. The drugs caused angiogenic inhibition in a small radius around the agarose pellets. The rest of the chorioallantoic membrane remained unaffected. This is a hint speaks for the specificity of angiogenesis inhibition and against a general cytotoxic effect on the embryo.

The software for the quantitative analysis generated solid data. The background signal was generally fairly high, since the segmentation routine had be set very sensitive to detect tiny vessel structures. This might lead to false positive signals suggesting a lower inhibition of angiogenesis than visually observed. The branching analyses were affected to a small extent by this issue. Therefore, only the total vessel areas were used in the fully established assay.

Comparison with an *ex ovo *chicken model showed good accordance of both test systems. Only the results for artesunate slightly differed from the results of the quail egg model. These differences were likely due to varying light conditions, resulting in a higher number of ghost structures in the artesunate treated quail eggs. This illustrates the importance of uniform illumination for correct quantification of the experiments. The main reason for switching from the well-established chicken egg CAM assay to quail eggs in the present approach was the size of the eggs. Due to the fact that quail eggs are smaller than chicken eggs, handling of eggs was facilitated. Less eggs were lost by damage during transport, the preparation of the *ex ovo* cultures was easier, and less space for the incubation of the eggs was required. 

### 4.2. Analysis of the Synergistic Interaction of Artesunate and Captopril

*In vitro* proliferation assays with human umbilical vein endothelial cells showed strong inhibition of proliferation by artesunate but not by captopril, although both substances inhibited angiogenesis. This suggests that they act by different mechanisms to inhibit angiogenesis. The results are in good accordance to previous reports. Artesunate directly inhibits proliferation and especially endothelial cell proliferation by VEGF inhibition [41]. Captopril on the other hand does not affect endothelial cell growth, but chemotaxis and capillary formation [35], effects which cannot be measured by proliferation assays. The wound-healing assay strongly supported the proposed mechanism of action. Artesunate led to remaining ruptures in the confluent cell monolayer, suggesting apoptotic or necrotic effects. On the other hand, captopril did not affect cell viability, but clearly inhibited migration of HUVECs in a dose-dependent manner.

Synergistic effects were found for captopril and artesunate *in vitro*. Combination treatments led to increased inhibition of wound-healing of up to 50% at a ratio of 60% captopril and 40% artesunate. The *ex ovo* CAM assay confirmed the synergism between both drugs *in vivo*. Taking together, we conclude that artesunate inhibited proliferation of endothelial cells and captopril inhibited capillary formation *via* chemotaxis. The cooperation of both mechanisms led to a synergistic inhibition of angiogenesis. While the CAM assay with quail eggs can be considered as a kind of *in vivo* assay, experiments in living animals are still missing and have to be done in the future to confirm the results obtained with HUVEC cells and quail eggs. 

To the best of our knowledge, the synergism of artesunate and captopril has been shown in the present investigation for the first time. This is a remarkable result, since artesunate is primarily a very effective antimalarial compound, which kills *Plasmodia* by free-radical production in the food vacuole and inhibition of a calcium ATPase in the parasites [42, 43]. This mechanism largely differs from other drugs that act synergistically in combination with captopril against cancer, such as the matrix metalloproteinase inhibitor, marimastat, or low molecular weight heparins [44]. 

Artesunate and captopril are both clinically established drugs. Artesunate is a safe malaria drug, which is commonly used to treat otherwise, drug-resistant *Plasmodium* strains. Captopril represents the lead compound for the class of angiotensin-converting enzyme (ACE) inhibitors to treat cardiovascular diseases. The toxicity of both drugs is rather low. Furthermore, artesunate has been described to exert profound anticancer activity against diverse human tumor types *in vitro* and *in vivo* [21–23]. Captopril is also known to inhibit tumor growth in mouse xenograft models [45]. The clinical use of captopril and artesunate and their synergistic interaction *in vivo* suggest the combination of both drugs to treat cancer in a clinical setting. Angiogenesis plays a key role in most solid cancer types providing a wide array of possible applications of artesunate/captopril combination therapy in clinical oncology.

Most anticancer drugs reveal severe toxicity with myelosuppression as one of the most critical ones. In this context, it is interesting that captopril ameliorates the hematological toxicity of doxorubicin [46]. Doxorubicin induces reactive oxygen species in addition to DNA intercalation and DNA topoisomerase II inhibition [47]. On the other hand, artesunate and other artemisinin-type drugs (which also produce reactive oxygen species) exert hematopoietic toxicity [48]. It is therefore worth speculating that a combination of artesunate and captopril does not only synergistically inhibit angiogenesis but might also lead to reduced side effects. This aspect warrants further investigation in the future. 

It is remarkable that ACE inhibitors which are in use for congestive heart failure and arterial hypertension for decades [33] also reveal anticancer activity. This may be of clinical relevance, since it has been reported that the use of ACE inhibitors is correlated with a lower incidence of skin cancer [49]. These results are supported by analyses on the molecular level, which revealed that ACE inhibitors including captopril reduce the expression of the vascular endothelial growth factor (VEGF) and RelA (NF-*κ*B) in tumors [35, 50–52]. Furthermore, ACE inhibitors downregulate matrix metalloproteinases, MMP-2, and MMP-9 and inhibit tumor metastasis [35, 53]. Angiotensin II represents a regulator of microvessel density, acting through the AT1 and AT2 receptors. Thereby, angiogenesis can be inhibited by ACE inhibitors such as captopril [54]. Interestingly, artesunate also inhibits tumor angiogenesis by downregulation of VEGF, KDR/flk-1, Flt-1, NF-*κ*B, and MMPs [21, 28, 55–57]. Furthermore, artesunate inhibits angiogenesis by induction of apoptosis in endothelial blood vessel cells [21, 58]. It can be speculated that the different antiangiogenic mechanisms of artesunate and captopril may act together in a complementary manner. This cooperative interaction of both drugs may generate the synergism observed in the present investigation. This hypothesis warrants further analyses. 

From a clinical point of view, the aspect of comorbidity of older patients is of interest. Cardiovascular diseases and cancer occur more frequently in geriatric patients. Therefore, patients suffering from cardiovascular symptoms who develop cancer may take advantage, if they also take captopril and artesunate at the same time to treat both diseases. The benefit of captopril-based combination treatments in comorbid patients have not been fully explored as yet, but several hints in the literature point to favorable treatment possibilities. Captopril has been reported to exert synergistic activity towards tumors in combination with cyclophosphamide [59], recombinant tissue plasminogen activator [60, 61], and synthetic metalloproteinase inhibitors such as batimastat [62] or marimastat [44]. 

In conclusion, the present investigation demonstrates the value of a combination cancer therapy of natural product derivatives originated from Chinese phytomedicine (artesunate) and snake venom (captopril). The results of this analysis merit further investigations addressing the question, whether not only artesunate but also extracts of *Artemisia annua* (e.g., as tea) synergistically interact with captopril to inhibit tumor angiogenesis and growth.
